# Melanin Photosensitization and the Effect of Visible Light on Epithelial Cells

**DOI:** 10.1371/journal.pone.0113266

**Published:** 2014-11-18

**Authors:** Orlando Chiarelli-Neto, Alan Silva Ferreira, Waleska Kerllen Martins, Christiane Pavani, Divinomar Severino, Fernanda Faião-Flores, Silvya Stuchi Maria-Engler, Eduardo Aliprandini, Glaucia R. Martinez, Paolo Di Mascio, Marisa H. G. Medeiros, Maurício S. Baptista

**Affiliations:** 1 Departamento de Bioquímica, Instituto de Química, Universidade de São Paulo, São Paulo, Brazil; 2 Departamento de Análises Clínicas, Faculdade de Ciências Farmacêuticas-USP, São Paulo, Brazil; 3 Departamento de Bioquímica e Biologia Molecular, Setor de Ciências Biológicas, Universidade Federal do Paraná, Curitiba, Brazil; Louisiana State University and A & M College, United States of America

## Abstract

Protecting human skin from sun exposure is a complex issue that involves unclear aspects of the interaction between light and tissue. A persistent misconception is that visible light is safe for the skin, although several lines of evidence suggest otherwise. Here, we show that visible light can damage melanocytes through melanin photosensitization and singlet oxygen (^1^O_2_) generation, thus decreasing cell viability, increasing membrane permeability, and causing both DNA photo-oxidation and necro-apoptotic cell death. UVA (355 nm) and visible (532 nm) light photosensitize ^1^O_2_ with similar yields, and pheomelanin is more efficient than eumelanin at generating ^1^O_2_ and resisting photobleaching. Although melanin can protect against the cellular damage induced by UVB, exposure to visible light leads to pre-mutagenic DNA lesions (i.e., Fpg- and Endo III-sensitive modifications); these DNA lesions may be mutagenic and may cause photoaging, as well as other health problems, such as skin cancer.

## Introduction

Humans and other animals produce melanin mainly for protection against exposure to ultraviolet B (UVB) radiation [1]. In contrast to UVB radiation, which is directly absorbed by DNA, UVA radiation acts essentially through photosensitization, which generates a triplet species, ^1^O_2_, and subsequently generates other radical species that can damage both DNA and other epithelial cell biomolecules [2]–[4]. UVA penetrates deeper in the dermis than does UVB, and it is the major UV source responsible for skin photoaging and the development of several types of skin cancer [5].

However, the visible portion of the spectra has garnered much less attention, despite several scientific reports that have described the effect of visible and IR irradiation on the skin [6], [7]. Kielbassa and co-workers [8] and Kvam and Tyrrel [9] showed that irradiating Chinese hamster cells and dermal fibroblasts, respectively, with UVA and visible light induced oxidative damage in DNA. More recent studies showed that visible light disturbs the epidermal barrier, and this disturbance induces pigmentation and inflammatory responses [10], [11]. However, a great deal of controversy remains concerning the effect of visible light on the skin, most likely because of the lack of a mechanism that explains the observed effects [12].

It has been shown that, in addition to UV [13], visible light also induces pigmentation in certain skin types. Mahmoud and co-workers [11] showed that visible light induces skin darkening in people with skin types IV and V but not in individuals with type II skin. The darkening induced by visible light depended on the pre-irradiation melanin content of the skin, suggesting that melanin may directly damage skin cells upon exposure to visible light.

The literature describes both protective and damaging roles for melanin [14], [15]. Two independent studies using *Xiphophorus*, which is a fish that is highly susceptible to melanoma, showed that the action spectra for both melanoma induction [16] and the photo-induced generation of reactive species [17] extend to visible wavelengths and that the shapes of the action spectra correspond to the shape of the melanin absorption spectrum. Despite the lack of a mechanistic explanation for the observations, these articles highlight the importance of understanding the role of the excited species that are generated after melanin is excited by visible light. In an earlier publication, we showed that melanin can act as a photosensitizer, leading to ^1^O_2_ generation after excitation with visible light [18]. Singlet oxygen can react with proteins, nucleic acids and membranes [19] (Figure S1); consequently, melanin photosensitization is likely involved in the phototoxicity of visible light, which is the main hypothesis that we aim to demonstrate herein.

## Methods

### Reagents

All solvents were spectroscopic grade. Water was distilled from an all-glass apparatus and further purified via a Millipore Milli-Q system. D_2_O (99%), tyrosine (Tyr), ammonium chloride (NH_4_Cl), the enzymes Fpg and Endo III, 3-(4,5-dimethylthiazol-2-yl)-2,5-diphenyltetrazolium bromide (MTT), KCl, Na_2_EDTA, HEPES and BSA from Sigma-Aldrich (either USA or Germany) were used as received. All other materials were the best analytical grade available. NaOD was prepared using three cycles of dissolution and evaporation of the initial NaOH solid (1 g) with D_2_O (10 g). The naphthalene derivative endoperoxide (DHPNO_2_) was synthesized by photosensitizing the precursor *N, N*′-di(2,3-dihydroxypropyl)-1,4-naphthalenedipropanamide (DHPN) with methylene blue as described by C. Pierlot et al [20]. The endoperoxide concentration was determined for each aliquot after the final filtration. The solutions were obtained at concentrations between 170 to 190 mmol/L with a purity of approximately 84%. This purity value refers to the amount of DHPNO_2_ obtained relative to the total amount of (DHPNO_2_+DHPN).

Eumelanin and pheomelanin were synthesized as described by Haywood et al with modifications [21]. Eumelanin was prepared from L-tyrosine (2.5 mg/mL) in pH 7.4 phosphate buffer (50 mM) and mushroom tyrosinase (150 U/mL) in bovine serum albumin (BSA) solution (5 mg/mL). Pheomelanin was synthesized from L-dopa (0.5 mg/mL) and L-cysteine (1.5 mg/mL) in pH 7.4 phosphate buffer (50 mM) and mushroom tyrosinase (100 U/mL). The reactions were performed at room temperature with stirring for 24 h. We also used eumelanin and pheomelanin samples that were kindly provided by Dr. S. Ito [22]. The samples from our lab and those from Dr. Ito’s lab behaved identically with regard to ^1^O_2_ generation.

### Equipment

The visible and UV light irradiator (Novatecnica, Brazil) included temperature and humidity sensors. Both variables were maintained during the experiments. The irradiation (mW/cm^2^) was measured at eight different areas in this irradiator using a dosimeter (VLX-3W, France). The irradiances of the light source were 3.0 mW.cm^−2^ and 3.3 mW.cm^−2^ in the UVB and visible regions, respectively. For UVB, 25 min of irradiation provided 4.5 J.cm^−2^. For the visible region, 30 min of irradiation provided 6 J.cm^−2^, 180 min of irradiation provided 36 J.cm^−2^, and 360 min of irradiation provided 72 J.cm^−2^.

Cell absorption/emission was quantified using a plate reader (Tecan Infinite 200M USA). The ^1^O_2_ measurements were performed in a specially designed instrument [18], [19], [23] consisting of a Surelite III laser (355 nm and 532 nm, 5-ns pulses, 10 pulses/s, 1 mJ/pulse; Continuum Lasers), cuvette holder, silicon filter, monochromator, liquid-nitrogen-cooled near infrared photomultiplier tube (NIR-PMT R5509) from Hamamatsu (Hamamatsu Co., Bridgewater, NJ, USA) and a fast multiscaler analyzer card with a resolution of 5 ns/channel (MSA-300; Becker & Hickl, Berlin, Germany). The signal was acquired either from a cell cuvette or directly under a fluorescence microscope (Nikon Eclipse Ti, USA). Fluorescence/transmission microscopy images were acquired from an Axiovert 200 microscope or an LSM 510 laser confocal microscope (Zeiss, Germany). The comet assay images were obtained using fluorescence microscopy (Olympus BH-2, USA). ImageJ Launcher was used for the confocal image analyses (National Institutes of Health, Bethesda).

### Cell culture

Several cell lines, which are available commercially, were received as gift: B16–F10 [24]; HaCaT [25]; J774 [26] and SK-mel 28 [27]. B16-F10, HaCaT and Hela cells were cultivated in Dulbecco’s Eagle (DMEM) culture medium (Sigma-Aldrich). J774 and SK-mel cells were cultivated in RPMI 1640 culture medium (Sigma-Aldrich). Both media were supplemented with 10% SFB (Gibco/BRL Life Technologies), 4 mM L-glutamine (Sigma USA), 100 U/mL penicillin (Sigma USA) and 100 mg/mL of streptomycin (Nissui Seiyaku) and incubated at 5% CO_2_ and 37°C. Primary skin cell cultures (melanocytes) were obtained from the foreskins of University Hospital (Hospital Universitário – HU-USP) patients [28]. The project was reviewed and approved by the Research Ethics Committee of the University Hospital (Av. Prof. Lineu Prestes, 2.565-Cidade Universitária-CEP 05508-000; +(5511)30919200, São Paulo, Brazil) (protocol# 943/09). The experiments were performed with each subject’s understanding and written consent, and the study methodologies conformed to the standards set by the Declaration of Helsinki. The melanocytes were maintained in 254CF medium (SKU# M-500-254CF; Cascade Biologics, USA) with human melanocyte growth supplement (HMGS – SKU# S-002-5; Cascade Biologics, USA).

### Melanogenesis, irradiation and viability

The cells were plated (2×10^4^ cells.mL^−1^) and, after 24 h, were treated with 0.5 mM Tyr (Sigma-Aldrich, Germany) and 10 mM NH_4_Cl (Labsynth, Brazil) for 48 h. This protocol increases the melanin production of melanocytes, and the resulting melanocytes and are referred to as M++++ herein; the control cells are referred to as CT. The cells were irradiated in PBS (8 g/L NaCl, 0.20 g/L KCl, 1.15 g/L Na_2_HPO_4_, and 0.2 g/L KH_2_PO_4_). We applied 4.5 J.cm^−2^ of UVB irradiation and 36 J.cm^−2^ and 72 J.cm^−2^ of visible irradiation. The cell density was evaluated using acridine orange fluorescence (excitation 488 nm, emission 515 nm). The cell viability was evaluated using MTT colorimetric and crystal violet assays [29]. Damage to the cytoplasmic membrane was quantified using propidium iodide incorporation. Apoptotic cell death was characterized using caspase-3 activation (Cell Signaling Technology, USA).

### B16-F10 cell viability after 24 h of a DHPNO_2_ treatment

The B16-F10 cells (CT and M++++) were treated with a solution containing RPMI and 10 mM DHPNO_2_ in the absence of serum for 2 h. In control assays, the cells were treated with a solution containing RPMI and 10 mM DHPN (decomposition product of DHPNO_2_) in the absence of serum for the same time period. After this period, the cell medium was changed to a normal culture medium (RPMI 1640 (Cultilab) supplemented with fetal calf serum (7.5%) from Gibco and with sodium bicarbonate (0.8 mM), HEPES (20 mM), and gentamicin (50 ng/mL) from Sigma-Aldrich. Cell viability was determined using an MTT assay 24 h after the DHPNO_2_ treatment.

### Melanin quantification

The melanin content was quantified as previously described [30]. The B16-F10 cells were seeded in 96-well plates (1×10^4^ cells.mL^−1^), and after 24 h, they were treated with 0.5 mM Tyr (Sigma-Aldrich Germany) and 10 mM NH_4_Cl (Labsynth Brazil) for 48 h. After incubation, a portion of the cells was centrifuged and suspended in 1 M NaOH (Labsynth Brazil). The other portion was maintained in PBS (8 g/L NaCl, 0.20 g/L KCl, 1.15 g/L Na_2_HPO_4_, and 0.2 g/L KH_2_PO_4_) for protein quantification. Both aliquots were lysed in a Branson Sonifier 450 (USA) at 20 W for 30 sec. The melanin was quantified by measuring the absorption at 470 nm in a Tecan Infinite 200M plate reader using a standard curve for commercial melanin (Sigma Aldrich Germany) [30]. The total protein content was determined using the Bradford method [31]. Melanin is expressed as µg of melanin/mg of protein.

### Comet assay

Comet assays were performed according to optimized protocols [32], [33]. B16-F10 cells were plated at 1×10^5^ cells.mL^−1^ in culture medium. Twenty-four hours after seeding, the cells were treated with 0.5 mM Tyr (Sigma-Aldrich, Germany) and 10 mM NH_4_Cl (Labsynth, Brazil) for 48 h. After incubation, the cells were irradiated in PBS (8 g/L NaCl, 0.20 g/L KCl, 1.15 g/L Na_2_HPO_4_, and 0.2 g/L KH_2_PO_4_) using 36 J.cm^−2^ or 6 J.cm^−2^ of visible light. We mixed 30 µl of a 1×10^5^ cell suspension with 100 µl of agarose (Sigma USA) (0.5% concentration in PBS) and distributed the mixture on slides that were pre-coated with agarose (Sigma USA) (1.5% concentration in PBS) and incubated on ice. After solidifying, the cells were lysed in the dark using a high-salt alkaline buffer (0.5 M NaCl, 0.1 M EDTA, 0.01 M Tris, and 1% Triton X-100, pH 10). For samples irradiated with 36 J.cm^−2^ or 6 J.cm^−2^, the slides were placed in electrophoresis buffer (0.3 M NaOH and 1 mM EDTA, pH 13, cooled in a refrigerator) in the dark for 30 min. Electrophoresis was performed in a cold-storage room, in the dark, using a power supply (ESP 301; GE USA) with the same buffer for 30 min at 25 V. After electrophoresis, the slides were neutralized using 0.4 M Tris at pH 7.5 and fixed in ethanol. In the protocol used to evaluate the direct DNA damage from melanin photosensitization, the cells were treated with 6 J.cm^−2^ of visible light, and before electrophoresis, the slides were treated with 0.2 U of Fpg or Endo III enzymes (Sigma-Aldrich, USA) in buffer (0.1 M KCl, 0.5 mM Na_2_EDTA, 40 mM HEPES and 0.2 mg/mL BSA at pH 8.0) for 30 minutes at 37°C. Subsequently, the DNA was stained with ethidium bromide (10 µg.mL^−1^), excited at 515 nm and observed using a fluorescence microscope (Olympus BH-2, USA) [34].

### ^1^O_2_ generation

The B16-F10 cells were seeded in six-well plates (2×10^5^ cells.mL^−1^), and after 24 h, they were treated with 0.5 mM Tyr (Sigma-Aldrich, Germany) and 10 mM NH_4_Cl (Labsynth, Brazil) for 48 h. The cells were washed in PBS (8 g/L NaCl, 0.20 g/L KCl, 1.15 g/L Na_2_HPO_4_, and 0.2 g/L KH_2_PO_4_), removed from the plates using a cell scraper and resuspended in D_2_O saline solution. We obtained ^1^O_2_ emission spectra by measuring the emission intensities from 1180 to 1360 nm with 1- to 5-nm steps using the equipment described above.

### Statistical analysis

The experiments were performed with least as three independent repetitions. The statistical analyses were assessed using Student’s t test and Microcal Origin software (version 7.0); P<0.05 was considered statistically significant.

## Results

**Figure 1:**
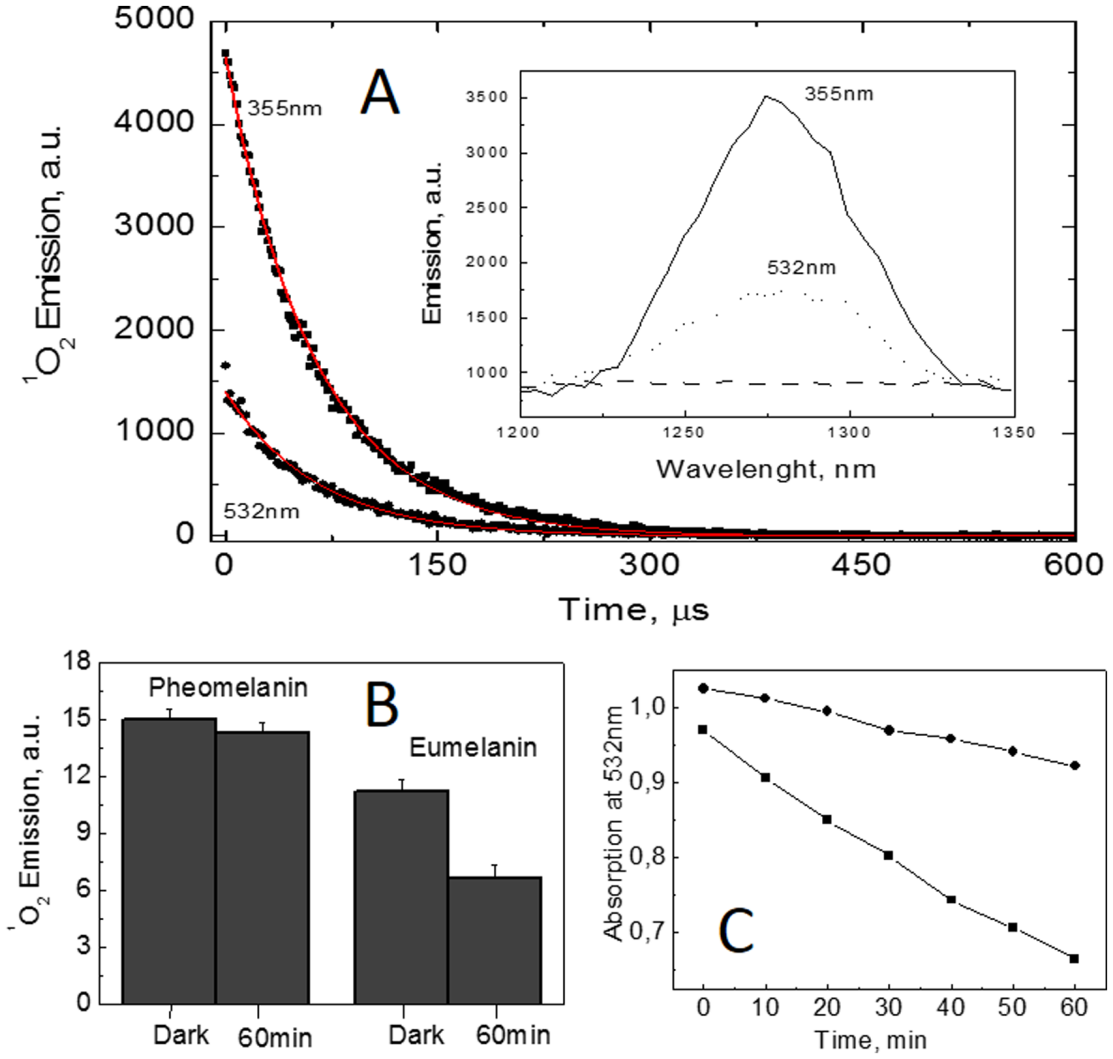
^1^O_2_ generation from melanin. (A) ^1^O_2_ decay from a eumelanin solution (0.04 g.L^−1^) in acetonitrile, pD = 10 with excitation at either 355 or 532 nm. The ^1^O_2_ decay lifetimes were the same under both conditions (∼17 µs, which is the lifetime expected for a mixture of acetonitrile and water) [18]. The insert shows the near infrared emission spectra after exciting a eumelanin solution at 532 nm and 355 nm in the absence of azide (continuous lines) and in the presence of 3 mM azide (dashed flat line); for the excitation pulses at both 355 nm and 532 nm, we used the following parameters: 5 ns, 10 pulses/s, and 1 mJ/pulse. (B) An integral of the emission spectra from pheomelanin (OD = 1.02) and eumelanin (OD = 0.97) solutions in acetonitrile immediately following dissolution (dark) and after 60 min of irradiation with visible light (light dose of 12 J.cm^−2^). (C) Absorption as a function of irradiation time for pheomelanin (•) and eumelanin (▪) solutions in acetonitrile.

The excitation of melanin with either UVA (355 nm) or visible (532 nm) light generated the characteristic ^1^O_2_ NIR emission spectra with a maximum centered at 1270 nm, which is a fingerprint of the O_2_ (a^1^Δg) → O_2_(X^3^Σg^−^) transition (Fig. 1). Sodium azide suppression confirmed this assignment (Fig. 1B, insert) [18], [19]. The emission intensity was stronger after melanin was excited by UVA than after excitation by visible light, but this difference is due to the greater melanin absorption at 355 compared with that at 532 nm; thus, the ^1^O_2_ generation efficiency does not vary depending on source of irradiation (UVA or visible).

Furthermore, the ^1^O_2_ emission from pheomelanin was 30% more intense than that from eumelanin (Fig. 1B), and visible light irradiation substantially decreased the absorption (Fig. 1C) and the generation of ^1^O_2_ by eumelanin (Fig. 1B); in contrast, the decrease (∼7%) in ^1^O_2_ generation by pheomelanin was substantially smaller (Fig. 1B). The chemical reaction underlying eumelanin photobleaching is the addition of ^1^O_2_ to the double bond at the C3 of the indole group with the consequent hydroperoxide formation [18]. This type of photoproduct was not detected from the pheomelanin photolysis.

**Figure 2:**
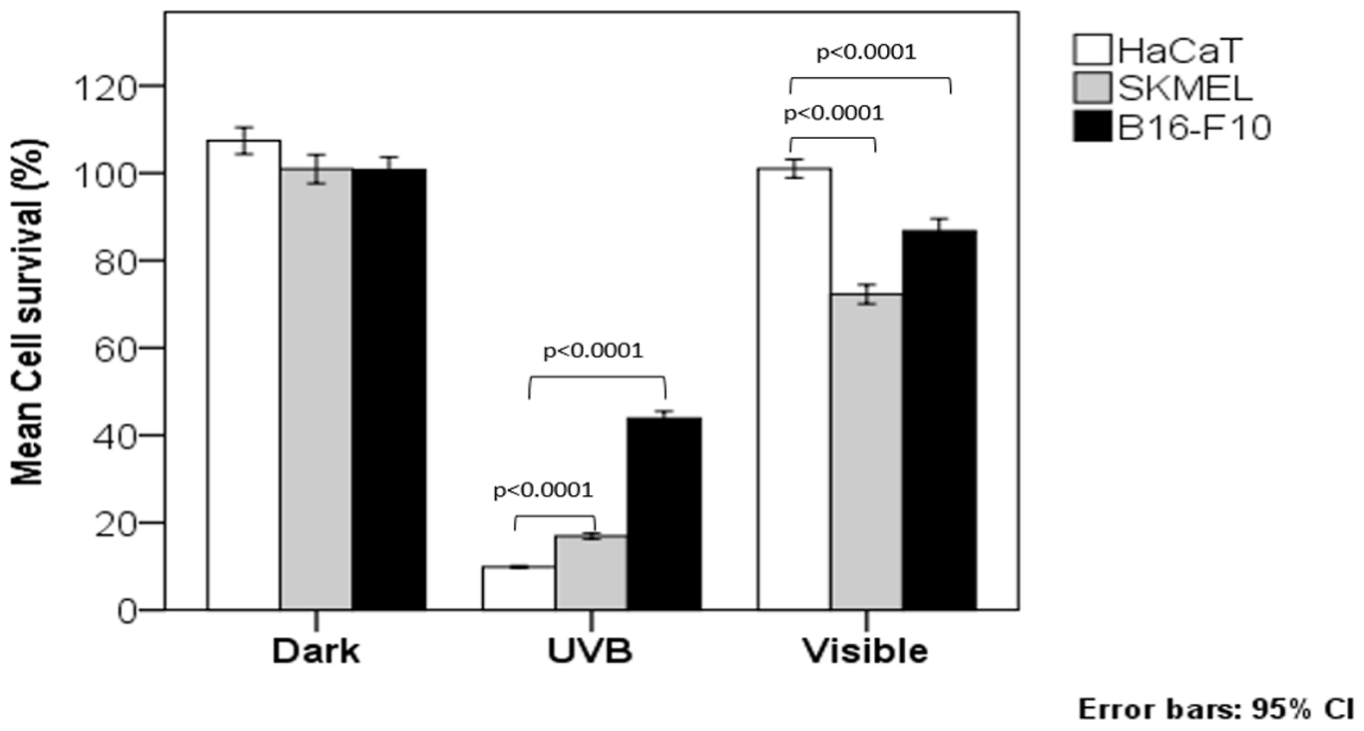
The effects of UVB and visible light on cell viability. The cell viability (MTT) of HaCaT, SK-mel and B16-F10 cells maintained in the dark (control) or irradiated with UVB (4.5 J.cm^−2^) or visible (36 J.cm^−2^) light. The color of the bars indicates the (qualitative) levels of melanin naturally produced by each cell line. Other cell lines, which are not melanocompetent (Hela, J774), were also tested and behaved similarly to the HaCaT cells (i.e., exhibited no phototoxicity from visible light at this dose (36 J.cm^−2^)).

To understand the potential effects of melanin photosensitization on epithelial cells, we compared the UVB and visible light photosensitivity using cell lines that express different amounts of melanin (Fig. 2). The irradiation doses were selected to mimic the exposure of an individual to ∼10 minutes of a sunny day in Brazil. Notably, the cells expressing more melanin had a higher survival rate after they were challenged with UVB, which is consistent with previous results [30]. However, the darker cells suffered from high phototoxicity from visible irradiation (Fig. 2), providing strong evidence that the phototoxicity from visible irradiation is related to the amount of melanin.

**Figure 3:**
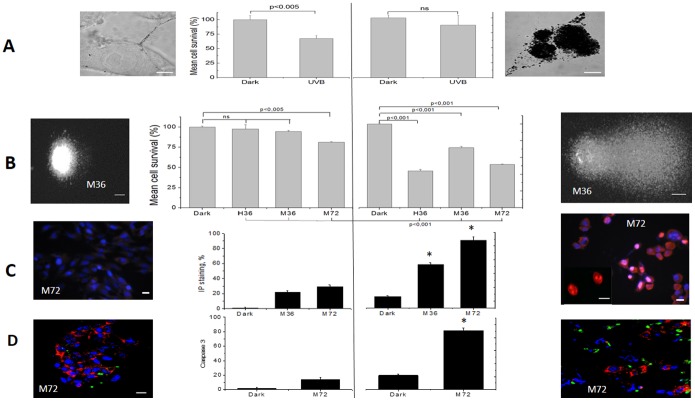
The effects of UVB and visible light in B16-F10 and human melanocyte cells. Left side: (no extra pigmentation); right side: cells subjected to the pigmentation protocol (M++++). (A) Viable cells (%) determined using the acridine orange fluorescence of B16-F10 cells that were maintained in the dark or with 4.5 J.cm^−2^ of UVB irradiation. The images are confocal optical microscopy images of the B16-F10 cells, CT (left) and M++++ (right). (B) The viable cells (%) were determined using the MTT colorimetric assay in the dark or with 36 J.cm^−2^ or 72 J.cm^−2^ of visible irradiation; murine B16-F10 cells are marked as either M36 or M72 depending on the light dose received. The human melanocytes (H36) only received a light dose of 36 J.cm^−2^. The images on the sides show comet assays performed using the CT and M++++ B16-F10 cells, 180 min after irradiation with a light dose of 36 J.cm^−2^ **.** (C) Propidium iodide incorporation in B16-F10 CT and M++++ cells in the dark and after treatment with 72 J.cm^−2^ of visible light. The images on the sides show typical images used for quantifying the PI incorporation. (D) Caspase 3 activation in B16-F10 CT and M++++ cells in the dark and after exposure to 72 J.cm^−2^ of visible light. The images on the side are typical for quantifying caspase 3 activation. (*) p<0.001.

We then tested the visible light toxicity in two melanocompetent cell lines (B16-F10 and human Caucasian melanocytes) under two different melanin production regimens (Fig. 3; i.e., basal level or control (CT) and induced melanogenesis (M++++)) [30]. Melanocytes in a culture clearly differ from skin melanocytes; thus, this system is a well-known, good experimental model for testing the cellular response to environmental challenges [35].

UVB exposure reduced the viability by 40% in B16-F10 CT (left), while pigmented cells (M++++, right) showed only a ∼9% reduction (Fig. 3). Therefore, the higher melanin content protected the pigmented cells from UVB damage, which is consistent with previous data from the literature [30], [36], [37]. The effect of visible light was opposite to the effect observed for UVB irradiation. Furthermore, upon irradiation with visible light (36 J.cm^−2^), the control cells for both the Caucasian melanocytes (H36) and the B16-F10 (M36) cells (left side, Fig. 3B) only showed a ∼5% decrease in cell viability. At 72 J.cm^−2^, the viability decrease was also small (i.e., 15%; M72, left side, Fig. 3B). However, when the cells were pigmented and treated with 36 or 72 J.cm^−2^ of visible light, both cell lines exhibited substantial decreases in viability (50% for H36, 25% for M36 and 40% for M72), which clearly demonstrates that the presence of melanin increases visible light phototoxicity. Moreover, the comet assay showed that the level of DNA fragmentation was higher in the pigmented cells than in the CT cells (Fig. 3B-images), which is consistent with increased visible light phototoxicity for the increased level of intracellular melanin. The mechanism of cell death was mainly necro-apoptosis; substantial levels of propidium iodide were incorporated (cytoplasmic membrane damage, Fig. 3C), and caspase 3 was activated (Fig. 3D).

**Figure 4:**
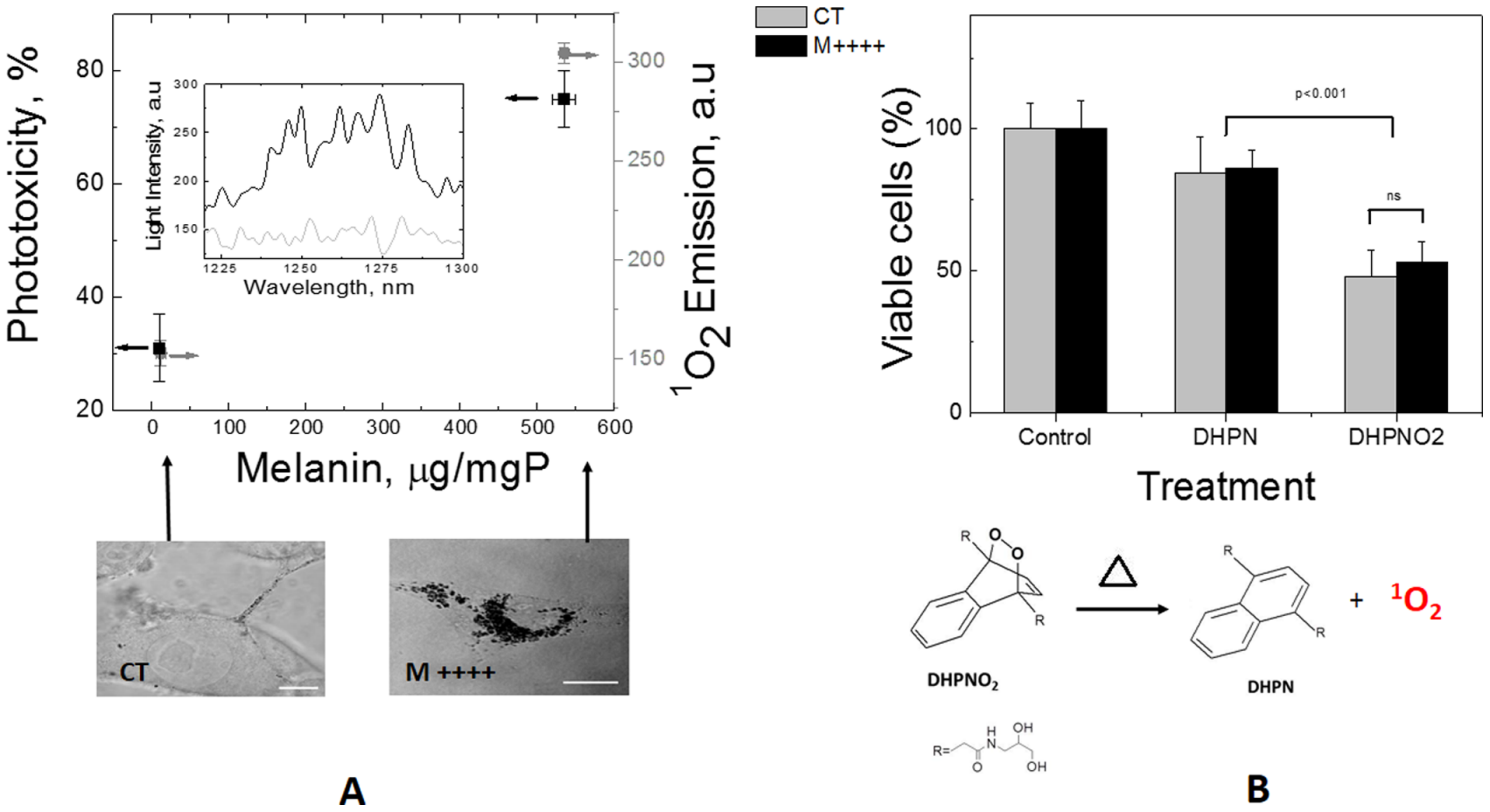
The effects of singlet oxygen on cells. (A) Phototoxicity and integrated emission spectra of the ^1^O_2_ that was generated upon excitation at 532 nm as a function of intracellular melanin production (in µg melanin/mg of total protein, squares) in B16-F10 CT or M++++ cells. Insert: ^1^O_2_ emission spectra from B16-F10 (CT and M++++) cells. The excitation wavelength was 532 nm 5-ns laser pulses at 10 pulses/s and 1 mJ/pulse. (B) Chemical generation of ^1^O_2_ by thermal degradation of DHPNO_2_ in CT and M++++ cells. The data are represented as the means±SD of three independent experiments, and the results are expressed as the percentage of viable cells compared with that in the control group. (*p<0.05 and ***p<0.001).

To correlate the visible light phototoxicity with the melanin and ^1^O_2_ contents, we quantified the amount of ^1^O_2_ generated in cells by measuring the near-infrared emission spectrum (1270 nm) after excitation with visible light (532 nm) [18] (Fig. 4A). The control cells only exhibited background signals (Fig. 4A, insert). However, the pigmented B16-F10 cells (M++++) showed the characteristic ^1^O_2_ spectra with a maximum intensity at approximately 1270 nm (the darker line in the Fig. 4A insert). Thus, the higher intracellular melanin content in the pigmented cells corresponded to more ^1^O_2_ generation and higher phototoxicity in response to visible irradiation compared with those for the control cells. To establish a definitive relationship between the ^1^O_2_ level and the cell toxicity, we also generated this species in the intracellular environment using a clean ^1^O_2_ source (i.e., thermal decomposition of DHPNO_2_). As shown in Fig. 4B, the cells treated with DHPNO_2_ exhibited a substantial decrease in viability (∼50%) compared with the viability of both types of control (cells without treatment and cells challenged with DHPN), which demonstrates that ^1^O_2_ plays a role in the reduced cell viability (Fig. 4B). Furthermore, the generation of ^1^O_2_ slightly reduced the viability of CT cells (49.7%) compared with that of M++++ cells (53.3%). This difference was not statistically significant, possibly because melanin can suppress ^1^O_2_. As reported earlier, melanin can exerts both types of effects, but in the presence of sufficient visible light illumination, melanin will stimulate the generation of ^1^O_2_ [14], [18], [38].

The excitation of melanin using visible light generates ^1^O_2_ and, consequently, the triplet species derived from melanin. Therefore, cellular damage can occur by both a type I mechanism (direct reaction between the triplet photosensitizer and biological targets, typically through an electron transfer reaction) and a type II mechanism (energy transfer reaction between the triplet photosensitizer and oxygen-forming ^1^O_2_) [19]. Depending on the severity of the damage, cell death will be the main outcome from visible light exposure. Another potential outcome, which is potentially more dangerous, is the generation of oxidative DNA products, which could lead to mutagenic compound accumulation, genomic instability and cancer [39].

**Figure 5:**
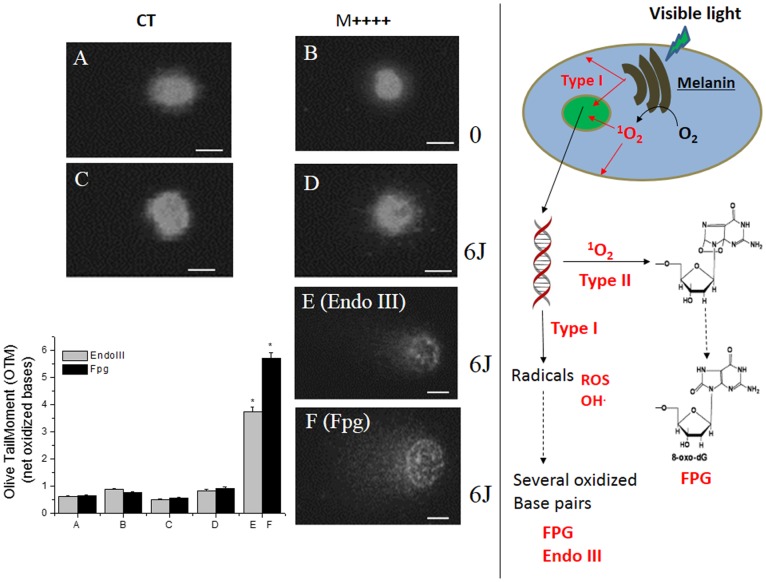
Melanin photosensitization with visible light damages nuclear DNA. (Left) Comet assay. (A) B16-F10 CT cells in the dark after treatment with 0.2 U of Fpg (treatment with Endo III yielded the same results); (B) B16-F10 M++++ cells in the dark after treatment with 0.2 U of Fpg (treatment with Endo III yielded the same results); (C) B16-F10 CT cells exposed to 6 J.cm^−2^ of visible irradiation after treatment with 0.2 U of Fpg (treatment with Endo III yielded the same results); (D) B16-F10 M++++ cells exposed to 6 J.cm^−2^ of visible irradiation without enzymes; (E, F) the same as D treated with 0.2 U of Endo III (E) and Fpg (F). The graphic on the left shows the quantification of the DNA fragmentation in B16-F10 cells under conditions A through E. (Right) A schematic of melanin photosensitization via type I and type II mechanisms, leading to damage in several biological targets, including the cell membrane and nuclear DNA. The DNA changes are indicated by using the main Fpg and Endo III recognition sites.

To demonstrate the direct damage to nuclear DNA by the melanin photosensitization that occurs in response to visible light irradiation, we performed a comet assay under low-dose conditions (i.e., a light dose that does not measurably decrease the cell viability for both CT and M++++ cells (6 J.cm^−2^)). After irradiation, the cells were treated with endonuclease enzymes (Fpg and Endo III) that recognize specific types of oxidative damage in DNA (Fig. 5). Fpg recognizes 8-oxoguanine, 8-hydroxyguanine and formamidopyrimidine, and Endo III recognizes strand breaks, abasic sites and additional oxidative pyrimidine modifications [40]. For a control, the comet assay was repeated in the absence of endonucleases. As expected, under this mild condition, both the CT and pigmented cells in the presence and absence of visible light did not show direct strand breaks, as indicated by the comet assay (data not shown, except for the M++++ cells with visible light irradiation, Fig. 5D). The first three controls (i.e., CT in the presence and absence of light and M++++ in the absence of light) also showed no DNA fragmentation after treatment with Fpg and Endo III (Fig. 5A–C). As mentioned above, the M++++ cells treated with light in the absence of these enzymes did not show DNA fragmentation (Fig. 5D). However, when Fpg or Endo III were applied to M++++ cells, which were irradiated with 6 J.cm^−2^ of visible light, the comet assay showed a considerable increase in the number of strand breaks (Fig. 5 graph and images E–F), which were absent in the controls (Fig. 5A–D). The presence of strand breaks demonstrated that melanin photosensitization by visible irradiation induces direct oxidative damage to nuclear DNA. The ratio of Fpg- to Endo III-sensitive modifications indicate that oxidative damage in DNA is most likely due to both type II and type I mechanisms [32], [33] (Fig. 5, scheme).

## Discussion

Although melanin is biosynthesized to protect against UVB, it will damage skin cells in the presence of UVA and visible light. At sufficiently high light doses, melanin causes extensive necro-apoptotic cell death. The lower light doses were able to mimic the potential chronic consequences of visible light exposure. Melanin induces the formation of pre-mutagenic DNA lesions (Fpg- and Endo III-sensitive modifications) (Fig. 4, scheme). We also showed that pheomelanin is a more efficient photosensitizer than eumelanin because it generates more ^1^O_2_ and better withstands photobleaching (i.e., it continuously generates ^1^O_2_ for longer periods than does eumelanin).

Therefore, in the presence of melanin, the effects of visible light irradiation do not differ from those of UVA irradiation; consequently, it should be considered with care, and further investigations must be performed to evaluate whether it may be a class I carcinogen [41]. These data indicate a causal relationship between visible light irradiation and the development of genome instability in melanocompetent cells and, consequently, the development of melanoma [17], [18], [36] and the higher skin cancer prevalence in individuals with red hair [15], [37]. Other authors have also concluded that visible light causes effects similar to those of UVA, such as inflammation and ROS production [6], [10], [17].

The current beliefs regarding the protection of skin against photoinduced damage are similar to those from 30 years ago but with a shift in the problematic wavelength region. In the early 1980 s, photobiologists knew that UVA induced cellular responses [2], [3]. However, people were convinced sunbathing with UVB-only protection was safe. The consequences of this strategy are felt today: the resulting deeper skin tumors have a higher prevalence of DNA mutations than those induced by UVA exposure [5], [42]. Clearly, visible light affects skin health, but people are encouraged to stay under the sun if they use sufficient amounts of “good sunscreen” (i.e., sunscreens that provide effective protection against UVA and UVB). This recommendation is clearly a mistake because it ignores the effects of visible light, which penetrates more deeply into skin than does UVB and UVA [43], and because it disregards the effects of other wavelength regions, such as the infrared region [44].

The toxicity of visible light raises concerns about other situations in addition to the direct sun phototoxicity to the skin, e.g., clinical protocols that use visible light, such as blue light therapy, in jaundiced babies [45]; indoor tanning [46]; the exposure of eyes to the toxicity of high levels of visible environmental light [47].

In the presence of melanin, visible light generates singlet oxygen and causes direct DNA damage. We hope this information will guide health professionals and the general population to safer interactions with the sun and, specifically, with visible light. We also hope that this information encourages companies to develop new sunscreen products that also provide protection against visible radiation.

## Supporting Information

Figure S1 **Scheme of the melanin photosensitization mechanisms that generate ^1^O_2_.** This ^1^O_2_ can react with the following to form several products: lipids mainly through an ene reaction that forms hydroperoxide, nucleic acids via a guanine residue to form 8-oxo-guanine, and amino acids (the scheme shows the amino acids that are most reactive with ^1^O_2_). The right side of the scheme shows the thermal decomposition of DHPNO_2_, which is also used to generate ^1^O_2_ in the intracellular environment.
(TIF)

## References

[1] Yamaguchi Y, Takahashi K, Zmudzka BZ, Kornhauser A, Miller SA, et al (2006) Human skin responses to UV radiation: pigment in the upper epidermis protects against DNA damage in the lower epidermis and facilitates apoptosis. FASEB journal: official publication of the Federation of American Societies for Experimental Biology 20: 1486–1488 Available: http://www.ncbi.nlm.nih.gov/pubmed/16793869 Accessed 2014 June 19. 16793869 10.1096/fj.06-5725fje

[2] Vile GF, Tyrrell RM (1995) UVA radiation-induced oxidative damage to lipids and proteins in vitro and in human skin fibroblasts is dependent on iron and singlet oxygen. Free radical biology & medicine 18: 721–730 Available: http://www.ncbi.nlm.nih.gov/pubmed/7750796 Accessed 2014 March 15. 7750796 10.1016/0891-5849(94)00192-m

[3] Berneburg M, Grether-Beck S, Kürten V, Ruzicka T, Briviba K, et al (1999) Singlet oxygen mediates the UVA-induced generation of the photoaging-associated mitochondrial common deletion. The Journal of biological chemistry 274: 15345–15349 Available: http://www.ncbi.nlm.nih.gov/pubmed/10336420 Accessed 2013 Dec 23. 10336420 10.1074/jbc.274.22.15345

[4] Cadet J, Douki T (2011) Oxidatively generated damage to DNA by UVA radiation in cells and human skin. The Journal of investigative dermatology 131: 1005–1007 Available: http://www.ncbi.nlm.nih.gov/pubmed/21494240 Accessed 2014 June 19. 21494240 10.1038/jid.2011.51

[5] Agar NS, Halliday GM, Barnetson RS, Ananthaswamy HN, Wheeler M, et al (2004) The basal layer in human squamous tumors harbors more UVA than UVB fingerprint mutations: a role for UVA in human skin carcinogenesis. Proceedings of the National Academy of Sciences of the United States of America 101: 4954–4959 Available: http://www.pubmedcentral.nih.gov/articlerender.fcgi?artid=387355&tool=pmcentrez&rendertype=abstract Accessed 2014 March 15. 15041750 10.1073/pnas.0401141101 PMC387355

[6] Grether-Beck S, Marini A, Jaenicke T, Krutmann J (2014) Photoprotection of human skin beyond ultraviolet radiation. Photodermatology, photoimmunology & photomedicine 30: 167–174 doi:10.1111/phpp.12111 10.1111/phpp.12111 24433486

[7] Mahmoud BH, Hexsel CL, Hamzavi IH, Lim HW (n.d.) Effects of visible light on the skin. Photochemistry and photobiology 84: 450–462 Available: http://www.ncbi.nlm.nih.gov/pubmed/18248499 Accessed 2014 Jan 19. 10.1111/j.1751-1097.2007.00286.x 18248499

[8] Kielbassa C, Roza L, Epe B (1997) Wavelength dependence of oxidative DNA damage induced by UV and visible light. Carcinogenesis 18: 811–816 Available: http://www.ncbi.nlm.nih.gov/pubmed/9111219 Accessed 2014 Jan 19. 9111219 10.1093/carcin/18.4.811

[9] Kvam E, Tyrrell RM (1997) Induction of oxidative DNA base damage in human skin cells by UV and near visible radiation. Carcinogenesis 18: 2379–2384 Available: http://www.ncbi.nlm.nih.gov/pubmed/9450485 Accessed 2014 Jan 19. 9450485 10.1093/carcin/18.12.2379

[10] Liebel F, Kaur S, Ruvolo E, Kollias N, Southall MD (2012) Irradiation of skin with visible light induces reactive oxygen species and matrix-degrading enzymes. The Journal of investigative dermatology 132: 1901–1907 Available: http://www.ncbi.nlm.nih.gov/pubmed/22318388 Accessed 2013 Nov 10. 22318388 10.1038/jid.2011.476

[11] Mahmoud BH, Ruvolo E, Hexsel CL, Liu Y, Owen MR, et al (2010) Impact of long-wavelength UVA and visible light on melanocompetent skin. The Journal of investigative dermatology 130: 2092–2097 Available: http://www.ncbi.nlm.nih.gov/pubmed/20410914 Accessed 2013 Nov 19. 20410914 10.1038/jid.2010.95

[12] Kolbe L (2012) How much sun protection is needed?: Are we on the way to full-spectrum protection? The Journal of investigative dermatology 132: 1756–1757 Available: http://www.ncbi.nlm.nih.gov/pubmed/22695285 Accessed 2014 Jan 19. 22695285 10.1038/jid.2012.148

[13] Tadokoro T, Yamaguchi Y, Batzer J, Coelho SG, Zmudzka BZ, et al (2005) Mechanisms of skin tanning in different racial/ethnic groups in response to ultraviolet radiation. The Journal of investigative dermatology 124: 1326–1332 Available: http://www.ncbi.nlm.nih.gov/pubmed/15955111 Accessed 2014 Jan 19. 15955111 10.1111/j.0022-202X.2005.23760.x

[14] Suzukawa AA, Vieira A, Winnischofer SMB, Scalfo AC, Di Mascio P, et al (2012) Novel properties of melanins include promotion of DNA strand breaks, impairment of repair, and reduced ability to damage DNA after quenching of singlet oxygen. Free radical biology & medicine 52: 1945–1953 Available: http://www.ncbi.nlm.nih.gov/pubmed/22401857 Accessed 2014 Jan 19. 22401857 10.1016/j.freeradbiomed.2012.02.039

[15] Tadokoro T, Kobayashi N, Zmudzka BZ, Ito S, Wakamatsu K, et al (2003) UV-induced DNA damage and melanin content in human skin differing in racial/ethnic origin. FASEB J 17: 1177–1179 Available: http://www.ncbi.nlm.nih.gov/pubmed/12692083. 12692083 10.1096/fj.02-0865fje

[16] Setlow RB, Grist E, Thompson K, Woodhead AD (1993) Wavelengths Effective in Induction of Malignant-Melanoma. Proceedings of the National Academy of Sciences of the United States of America 90: 6666–6670. Available: <Go to ISI>://A1993LM68100060. 10.1073/pnas.90.14.6666 PMC46993 8341684

[17] Wood SR, Berwick M, Ley RD, Walter RB, Setlow RB, et al (2006) UV causation of melanoma in Xiphophorus is dominated by melanin photosensitized oxidant production. Proceedings of the National Academy of Sciences of the United States of America 103: 4111–4115 Available: http://www.pubmedcentral.nih.gov/articlerender.fcgi?artid=1449655&tool=pmcentrez&rendertype=abstract Accessed 2014 March 15. 16537493 10.1073/pnas.0511248103 PMC1449655

[18] Chiarelli-Neto O, Pavani C, Ferreira AS, Uchoa AF, Severino D, et al (2011) Generation and suppression of singlet oxygen in hair by photosensitization of melanin. Free radical biology & medicine 51: 1195–1202 Available: http://www.ncbi.nlm.nih.gov/pubmed/21723388 Accessed 2013 Nov 18. 21723388 10.1016/j.freeradbiomed.2011.06.013

[19] Ogilby PR (2010) Singlet oxygen: there is indeed something new under the sun. Chemical Society reviews 39: 3181–3209 Available: http://www.ncbi.nlm.nih.gov/pubmed/20571680 Accessed 2014 April 4. 20571680 10.1039/b926014p

[20] Pierlot C, Aubry JM, Briviba K, Sies H, Di Mascio P (2000) Naphthalene endoperoxides as generators of singlet oxygen in biological media. Methods in enzymology 319: 3–20 Available: http://www.ncbi.nlm.nih.gov/pubmed/10907494 Accessed 2014 June 19. 10907494 10.1016/s0076-6879(00)19003-2

[21] Haywood RM, Lee M, Andrady C (2008) Comparable photoreactivity of hair melanosomes, eu- and pheomelanins at low concentrations: low melanin a risk factor for UVA damage and melanoma? Photochemistry and photobiology 84: 572–581 Available: http://www.ncbi.nlm.nih.gov/pubmed/18399925 Accessed 2014 Jan 19. 18399925 10.1111/j.1751-1097.2008.00343.x

[22] Ito S, Wakamatsu K (2003) Quantitative analysis of eumelanin and pheomelanin in humans, mice, and other animals: a comparative review. Pigment Cell Res 16: 523–531 Available: http://www.ncbi.nlm.nih.gov/pubmed/12950732. 12950732 10.1034/j.1600-0749.2003.00072.x

[23] Uchoa AF, Knox PP, Turchielle R, Seifullina Nk, Baptista MS (2008) Singlet oxygen generation in the reaction centers of Rhodobacter sphaeroides. Eur Biophys J 37: 843–850 Available: http://www.ncbi.nlm.nih.gov/pubmed/18286272. 18286272 10.1007/s00249-008-0287-y

[24] Fidler IJ (1975) Biological behavior of malignant melanoma cells correlated to their survival in vivo. Cancer research 35: 218–224 Available: http://www.ncbi.nlm.nih.gov/pubmed/1109790 Accessed 2014 Oct 9. 1109790

[25] Boukamp P, Petrussevska RT, Breitkreutz D, Hornung J, Markham A, et al (1988) Normal keratinization in a spontaneously immortalized aneuploid human keratinocyte cell line. The Journal of cell biology 106: 761–771 Available: http://www.pubmedcentral.nih.gov/articlerender.fcgi?artid=2115116&tool=pmcentrez&rendertype=abstract Accessed 2014 Sept 20. 2450098 10.1083/jcb.106.3.761 PMC2115116

[26] Ralph P, Nakoinz I (1975) Phagocytosis and cytolysis by a macrophage tumour and its cloned cell line. Nature 257: 393–394 Available: http://www.ncbi.nlm.nih.gov/pubmed/1101071 Accessed 2014 Oct 9. 1101071 10.1038/257393a0

[27] Carey TE, Takahashi T, Resnick LA, Oettgen HF, Old LJ (1976) Cell surface antigens of human malignant melanoma: mixed hemadsorption assays for humoral immunity to cultured autologous melanoma cells. Proceedings of the National Academy of Sciences of the United States of America 73: 3278–3282 Available: http://www.pubmedcentral.nih.gov/articlerender.fcgi?artid=431008&tool=pmcentrez&rendertype=abstract Accessed 2014 Oct 9. 1067619 10.1073/pnas.73.9.3278 PMC431008

[28] Brohem CA, Massaro RR, Tiago M, Marinho CE, Jasiulionis MG, et al (2012) Proteasome inhibition and ROS generation by 4-nerolidylcatechol induces melanoma cell death. Pigment cell & melanoma research 25: 354–369 Available: http://www.ncbi.nlm.nih.gov/pubmed/22372875 Accessed 2014 Oct 9. 22372875 10.1111/j.1755-148X.2012.00992.x

[29] Collier AC, Pritsos CA (2003) The mitochondrial uncoupler dicumarol disrupts the MTT assay. Biochemical pharmacology 66: 281–287 Available: http://www.ncbi.nlm.nih.gov/pubmed/12826270 Accessed 2014 Dec 23. 12826270 10.1016/s0006-2952(03)00240-5

[30] Heo S-J, Ko S-C, Cha S-H, Kang D-H, Park H-S, et al (2009) Effect of phlorotannins isolated from Ecklonia cava on melanogenesis and their protective effect against photo-oxidative stress induced by UV-B radiation. Toxicology in vitro: an international journal published in association with BIBRA 23: 1123–1130 Available: http://www.ncbi.nlm.nih.gov/pubmed/19490939 Accessed 2013 Nov 30. 19490939 10.1016/j.tiv.2009.05.013

[31] Bradford MM (1976) A rapid and sensitive method for the quantitation of microgram quantities of protein utilizing the principle of protein-dye binding. Analytical biochemistry 72: 248–254 Available: http://www.ncbi.nlm.nih.gov/pubmed/942051. 942051 10.1016/0003-2697(76)90527-3

[32] Pouget JP, Douki T, Richard MJ, Cadet J (2000) DNA damage induced in cells by gamma and UVA radiation as measured by HPLC/GC-MS and HPLC-EC and Comet assay. Chemical research in toxicology 13: 541–549 Available: http://www.ncbi.nlm.nih.gov/pubmed/10898585 Accessed 2014 June 19. 10898585 10.1021/tx000020e

[33] Sauvaigo S, Petec-Calin C, Caillat S, Odin F, Cadet J (2002) Comet assay coupled to repair enzymes for the detection of oxidative damage to DNA induced by low doses of gamma-radiation: use of YOYO-1, low-background slides, and optimized electrophoresis conditions. Analytical biochemistry 303: 107–109 Available: http://www.ncbi.nlm.nih.gov/pubmed/11906159 Accessed 2014 June 19. 11906159 10.1006/abio.2001.5559

[34] Olive PL, Banáth JP (2006) The comet assay: a method to measure DNA damage in individual cells. Nature protocols 1: 23–29 Available: http://www.ncbi.nlm.nih.gov/pubmed/17406208 Accessed 2014 Jan 10. 17406208 10.1038/nprot.2006.5

[35] Hearing VJ (2000) The melanosome: the perfect model for cellular responses to the environment. Pigment cell research/sponsored by the European Society for Pigment Cell Research and the International Pigment Cell Society 13 Suppl 8: 23–34 Available: http://www.ncbi.nlm.nih.gov/pubmed/11041354 Accessed 2014 June 19. 10.1034/j.1600-0749.13.s8.7.x 11041354

[36] Lin JY, Fisher DE (2007) Melanocyte biology and skin pigmentation. Nature 445: 843–850 Available: http://www.ncbi.nlm.nih.gov/pubmed/17314970 Accessed 2014 Jan 18. 17314970 10.1038/nature05660

[37] Rees JL (2003) Genetics of hair and skin color. Annual review of genetics 37: 67–90 Available: http://www.ncbi.nlm.nih.gov/pubmed/14616056 Accessed 2014 Jan 19. 10.1146/annurev.genet.37.110801.143233 14616056

[38] Beltrán-García MJ, Prado FM, Oliveira MS, Ortiz-Mendoza D, Scalfo AC, et al (2014) Singlet molecular oxygen generation by light-activated DHN-melanin of the fungal pathogen Mycosphaerella fijiensis in black Sigatoka disease of bananas. PloS one 9: e91616. Available: http://www.pubmedcentral.nih.gov/articlerender.fcgi?artid=3960117&tool=pmcentrez&rendertype=abstract. ccessed 2014 June 19. 10.1371/journal.pone.0091616 PMC3960117 24646830

[39] Russo MT, Blasi MF, Chiera F, Fortini P, Degan P, et al (2004) The oxidized deoxynucleoside triphosphate pool is a significant contributor to genetic instability in mismatch repair-deficient cells. Molecular and cellular biology 24: 465–474 Available: http://www.pubmedcentral.nih.gov/articlerender.fcgi?artid=303369&tool=pmcentrez&rendertype=abstract Accessed 2014 Jan 19. 14673178 10.1128/MCB.24.1.465-474.2004 PMC303369

[40] Hatahet Z, Kow YW, Purmal AA, Cunningham RP, Wallace SS (1994) New substrates for old enzymes. 5-Hydroxy-2′-deoxycytidine and 5-hydroxy-2′-deoxyuridine are substrates for Escherichia coli endonuclease III and formamidopyrimidine DNA N-glycosylase, while 5-hydroxy-2′-deoxyuridine is a substrate for uracil DNA N-glycos. The Journal of biological chemistry 269: 18814–18820 Available: http://www.ncbi.nlm.nih.gov/pubmed/8034633 Accessed 2014 Jan 19. 8034633

[41] Dahle J, Kvam E (2003) Induction of delayed mutations and chromosomal instability in fibroblasts after UVA-, UVB-, and X-radiation. Cancer research 63: 1464–1469 Available: http://www.ncbi.nlm.nih.gov/pubmed/12670891 Accessed 2014 Jan 19. 12670891

[42] Halliday GM, Agar NS, Barnetson RSC, Ananthaswamy HN, Jones AM (2005) UV-A fingerprint mutations in human skin cancer. Photochemistry and photobiology 81: 3–8 Available: http://www.ncbi.nlm.nih.gov/pubmed/15335275 Accessed 2014 March 15. 15335275 10.1562/2004-07-27-IR-247

[43] Mahmoud BH, Hexsel CL, Hamzavi IH, Lim HW (2007) Effects of visible light on the skin. Photochemistry and photobiology 84: 450–462 Available: http://www.ncbi.nlm.nih.gov/pubmed/18248499 Accessed 2014 March 6. 10.1111/j.1751-1097.2007.00286.x 18248499

[44] Schieke SM, Schroeder P, Krutmann J (2003) Cutaneous effects of infrared radiation: from clinical observations to molecular response mechanisms. Photodermatology, photoimmunology & photomedicine 19: 228–234 Available: http://www.ncbi.nlm.nih.gov/pubmed/14535893 Accessed 2014 March 16. 10.1034/j.1600-0781.2003.00054.x 14535893

[45] Opländer C, Hidding S, Werners FB, Born M, Pallua N, et al (2011) Effects of blue light irradiation on human dermal fibroblasts. Journal of photochemistry and photobiology B, Biology 103: 118–125 Available: http://www.ncbi.nlm.nih.gov/pubmed/21421326 Accessed 2014 Jan 19. 10.1016/j.jphotobiol.2011.02.018 21421326

[46] Youssef PN, Sheibani N, Albert DM (2011) Retinal light toxicity. Eye (London, England) 25: 1–14 Available: http://www.pubmedcentral.nih.gov/articlerender.fcgi?artid=3144654&tool=pmcentrez&rendertype=abstract Accessed 2014 Jan 19. 10.1038/eye.2010.149 PMC3144654 21178995

[47] Fisher DE, James WD (2010) Indoor tanning–science, behavior, and policy. The New England journal of medicine 363: 901–903 Available: http://www.pubmedcentral.nih.gov/articlerender.fcgi?artid=3951814&tool=pmcentrez&rendertype=abstract Accessed 2014 March 16. 20818900 10.1056/NEJMp1005999 PMC3951814

