# Supplementary figures and images for: Melanin Photosensitization and the Effect of Visible Light on Epithelial Cells
Source: PLoS One. 2014 Nov 18;9(11):e113266. doi: 10.1371/journal.pone.0113266 (PMC4236153; doi:10.1371/journal.pone.0113266)

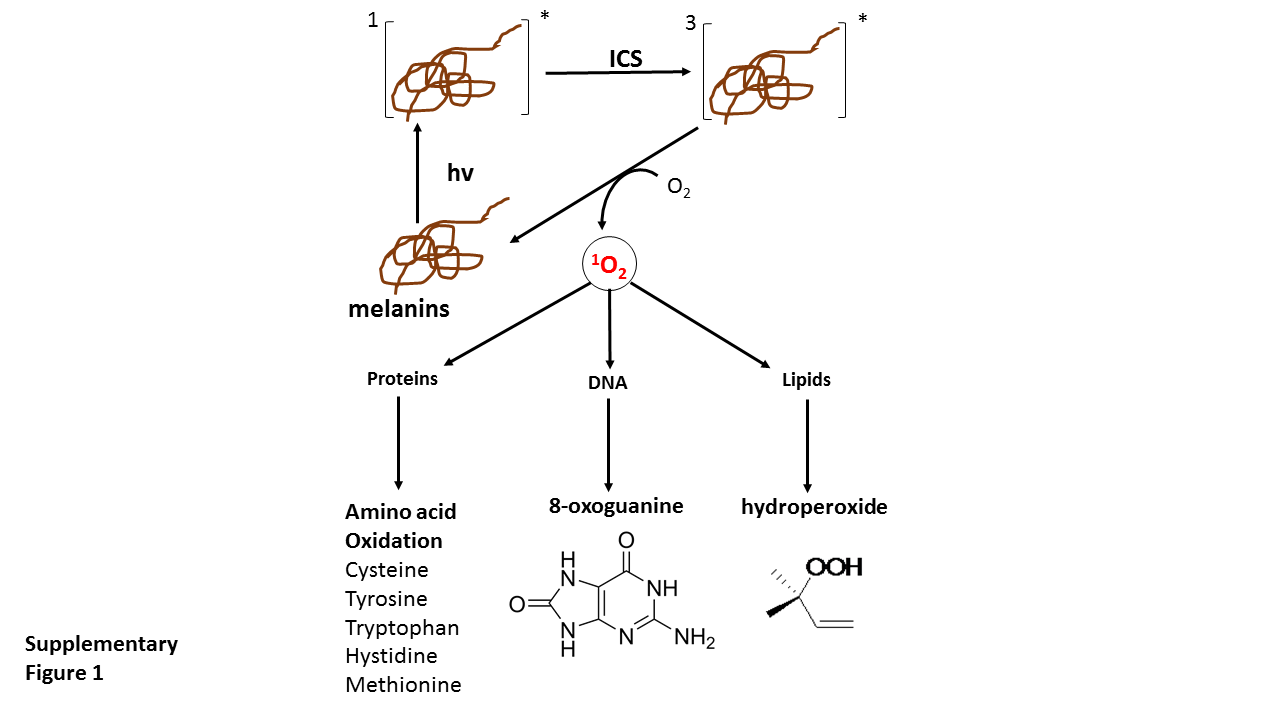

Supplement: Figure S1 — Scheme of the melanin photosensitization mechanisms that generate 1O2. This 1O2 can react with the following to form several products: lipids mainly through an ene reaction that forms hydroperoxide, nucleic acids via a guanine residue to form 8-oxo-guanine, and amino acids (the scheme shows the amino acids that are most reactive with 1O2). The right side of the scheme shows the thermal decomposition of DHPNO2, which is also used to generate 1O2 in the intracellular environment. (TIF) [file pone.0113266.s001.tif]
